# Could Horse Gait and Induced Pelvic Dynamic Loads in Female Equestrians Be a Risk Factor in Pudendal Neuralgia?

**DOI:** 10.3390/sports11010016

**Published:** 2023-01-10

**Authors:** Sébastien Murer, Guillaume Polidori, Fabien Beaumont, Fabien Bogard, Hassen Hakim, Fabien Legrand

**Affiliations:** 1 MATIM, Université de Reims Champagne-Ardenne, UFR Sciences Exactes et Naturelles, Campus Moulin de la Housse, 51100 Reims, France; 2 BMBI-UMR CNRS 7338, Centre de Recherches de Royallieu, Université de Technologie de Compiègne, 60200 Compiègne, France; 3 Laboratory of Exercise Physiology and Physiopathology, Faculty of Medicine, University of Sousse Ibn Jazzar, Sousse 4002, Tunisia; 4 C2S, Department of Psychology, Université de Reims Champagne-Ardenne, 51100 Reims, France

**Keywords:** dynamic horseback riding, saddle–rider interaction, pelvic floor trauma, pressure mapping

## Abstract

Pudendal Neuralgia (PN) is a rare, debilitating disease caused by damage to the pudendal nerve, which innervates the anus, rectum, perineum, lower urinary tract, and genitalia. Although its etiology remains scientifically unknown, a number of sports practices, including horse-riding, are reported as triggering and/or aggravating factors. The present work summarizes the experimental measurements of the contact pressure at the interface between the rider and saddle, for a population of 12 experienced female riders. These tests reveal that dynamic horseback-riding leads to high levels of peak pressures in the perineal region, which confirms that the practice of equine sports may cause neuropathologies such as PN. All collected data will be used as boundary conditions in a future numerical 3D model aimed at locating the possible areas of pudendal nerve crushing.

## 1. Introduction

Pudendal neuralgia (PN) is a rare pathology arising from damage to the pudendal nerve in Alcock’s canal. The pudendal nerve emerges from segments S2 to S4, then passes between the sacrospinous and sacrotuberous ligaments, before running through the lesser sciatic foramen. It is at this point that it enters the L-shaped pudendal canal (sometimes referred to as “Alcock’s canal”) and passes over the falciform process of the sacrotuberous ligament. In females, the nerve splits into three main branches (the dorsal nerve of the clitoris, the perineal nerve and the inferior rectal nerve) and is also responsible for innervation of the perineum and its superficial muscles and skin. As a whole, this specific path makes the pudendal nerve anatomically vulnerable to compression and entrapment.

PN interferes with various primary biologic functions such as urination, defecation, and sexual activity, and it reduces the ability to maintain a number of body postures or to simply move around. As mentioned by Labat et al. [1], PN is manifested through various symptoms such as burning, torsion, or striction sensations, electrical shocks of varying intensities, and/or sensitivity disorders. The pain is most often unilateral and increases with sitting [2]. In all cases, the patient’s quality of life is severely degraded.

PN was first described by Zuelzer in 1915, but it was only in 1987 that a paper by Amarenco et al. [3] drew attention on the disease. Yet, to date, diagnosis remains misunderstood, PN remains little or not addressed in medical schools, and the patients often do not dare seek medical attention. The International Pudendal Neuropathy Foundation reports an incidence rate of 1/100,000, with a prevalence in women [4] that oriented the choice of test subjects in the present study. Most of the patients are also aged between 50 and 70, and various therapeutic approaches exist, although no efficacy-based recommendations exist [5].

**Figure 1:**
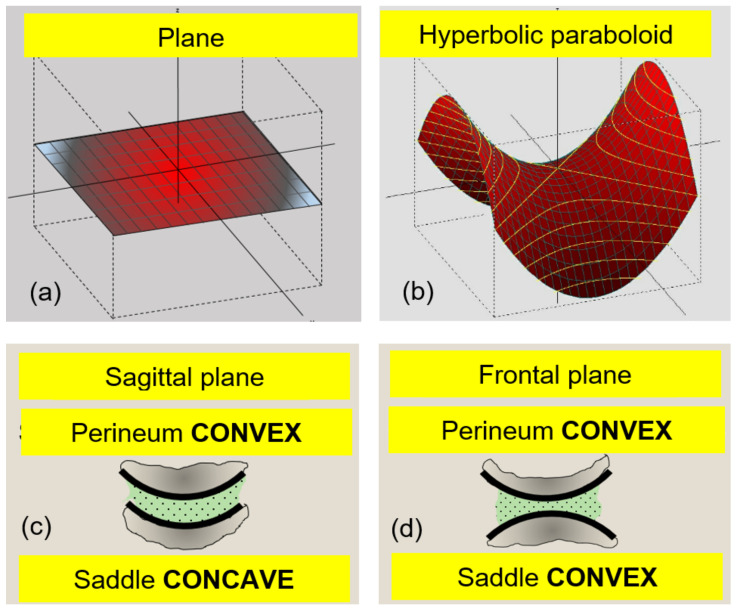
Synopsis of the planar and saddle geometries (**a**,**b**), and the interactions between the saddle and the rider’s perineum in the sagittal (**c**) and frontal (**d**) planes.

Regarding the etiology of PN in sports, many articles in the literature mention the practice of cycling as a triggering or aggravating factor, especially in long-distance practice [6,7,8,9,10,11,12]. Similarly, horse-riders are more susceptible to compression injuries, since horseback-riding involves cyclic solicitations over the perineal area. This practice has also been reported as a possible cause for PN and related pathologies [6,13,14,15]. Yet, to the best of the authors’ knowledge, no study seems to have focused on the mechanical actions occurring at the rider-saddle interface, especially about the resulting pressure levels. Together with the saddle’s shape and the rider’s weight, the horse’s movement affects the distribution of interfacial pressure, which can lead to undesirable effects such as tissue compression and shearing, or traumatic micro-shock of the rider’s perineal area. Classical seating surfaces (Figure 1a) display limited curvatures, and have been the subject of a number of studies [16,17,18,19]. Conversely, the saddle of a horse has a complex geometry (Figure 1b), with a hyperbolic paraboloid shape, both concave in the sagittal plane of the rider and convex in its frontal plane. In a simplified way, it can be considered that the perineum results in a convex shape, both in its sagittal and frontal planes (Figure 1c,d).

**Figure 2:**
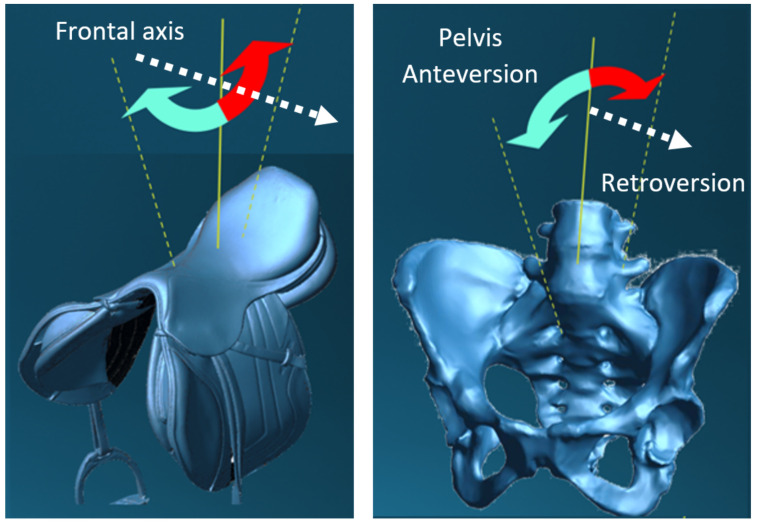
Contra-rotative motions of pelvis and saddle in canter and gallop horseback-riding.

To this initial complexity of interaction between the geometry of the saddle and the perineum, are added the rotating movements of the horse and rider around the frontal axis, in phase opposition (Figure 2).

**Figure 3:**
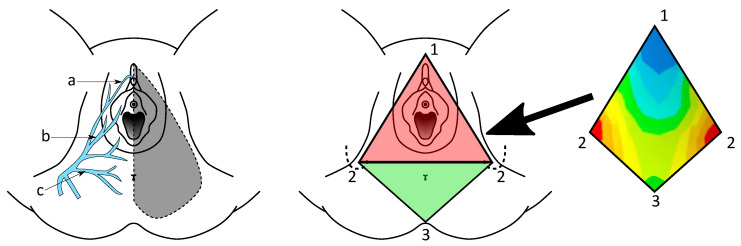
(**Left**) Ramifications of the pudendal nerve: dorsal nerve of the clitoris (a), perineal nerve (b), inferior rectal nerve (c), and pudendal nerve territory (shaded); (**Center**) Perineum anatomy: urogenital (red) and anal (green) triangles with their vertices, i.e., pubic symphysis (1), ischial tuberosities (2), and apex of the sacrum (3). (**Right**): Typical pressure mapping over the perineum area. Original artwork.

These counter-rotating movements will successively and cyclically affect the anterior urogenital triangle delimited by the pubic symphysis and the ischial tuberosities, as well as the posterior anal triangle extending to the apex of the sacrum. These different mechanical loads affect the entire perineal area, which is also the territory of the pudendal nerve, the main nerve of the perineum (see Figure 3), but more importantly, there is never optimal relief of the sensitive structures of the perineal area. Thus, obtaining mechanical data should allow for a better understanding of the physical phenomena that can lead to pudendal nerve entrapment.

Therefore, the works described herein aim at collecting data on the distribution of contact pressure over the pudendal nerve territory, in a population of 12 confirmed female horse riders. For the sake of simplicity and in an attempt to limit non-replicability bias, an electromechanical horse simulator was used throughout the experiments.

## 2. Materials and Methods

As PN mostly affects females, 12 healthy female riders with at least five years of practice were selected and agreed to participate in the study. None of them reported signs of PN. They were asked to give informed consent prior to the test campaign. The experimental setup mainly consisted of an electromechanical horse simulator (P. Klavins, Le Simulateur Équestre Français, Changé, France), which the participants were familiar with. A flexible pressure mattress (TexiSense Teximat^®^; 32×32 sensors; spatial resolution 14.7 mm; accuracy ±10%, range 0.3–45 kPa), validated in previous studies [16,20,21], was placed between the saddle and the rider. It should be noted that only the normal component of stress was captured. However, the pudendal nerve territory is mostly horizontal, and hence, perpendicular to the body weight direction: it can be reasonably assumed that the contact pressure was properly measured over the area of interest. Each measurement was carried out for a duration of 30 s, which guaranteed the stabilization of the cycle. In a second step, the pressure peaks (i.e., maximum pressure values) in the pudendal nerve territory, as well as the location of the center of pressure (CoP), were extracted using the TexiMonitor^®^ software.

Three riding situations are investigated for each of the 12 participants. One is static (simulator halted), and the other two are dynamic for canter and gallop, at simulator frequencies of 0.8 Hz (“slow dynamic”) and 2 Hz (“fast dynamic”), respectively. Pressure distributions are recorded at a sampling rate of 10 Hz, which ensures proper acquisition, given the operating frequencies.

Statistical analysis is performed using a t-test on all 12 values obtained in the different situations (stationary, slow dynamic, fast dynamic, and anterior and posterior phases).

## 3. Results

**Figure 4:**
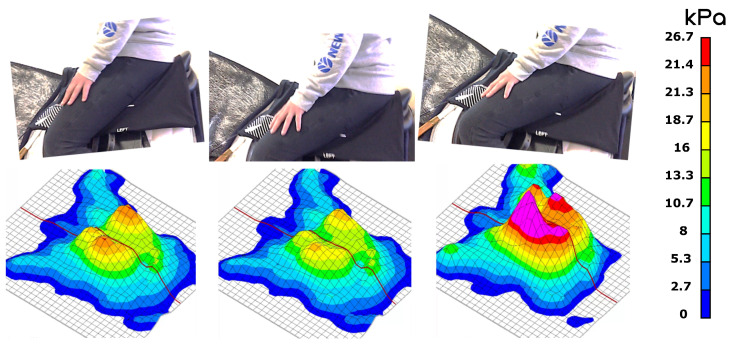
Pressure mapping during a slow dynamic test: posterior (**left**), centered (**middle**), and anterior (**right**) phases.

Figure 4 presents a set of experimental pressure distributions over a simulator cycle. Three characteristic phases, determined by a visual assessment of the raw data sequence, may be distinguished: the posterior phase (left), where the simulator is tilted forward, the horizontal phase (center), where its direction is parallel to the ground, and the anterior phase (right), where the simulator is tilted backward.

**Figure 5:**
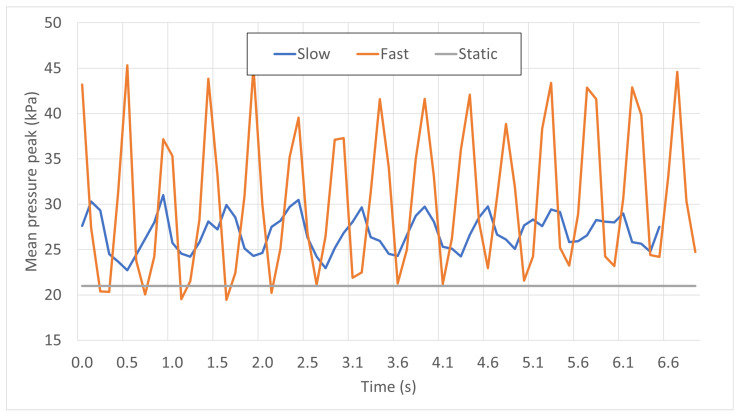
Time-dependent mean pressure peaks in static, slow, and fast dynamic situations.

Based on similar measurements, the time-dependent pressure peaks in stationary, slow, and fast dynamic conditions are averaged over all 12 participants, and plotted in Figure 5. Individual curves were first synchronized and a time frame of around 7 s, which is sufficient to observe the stability of the solicitations, was selected.

**Figure 6:**
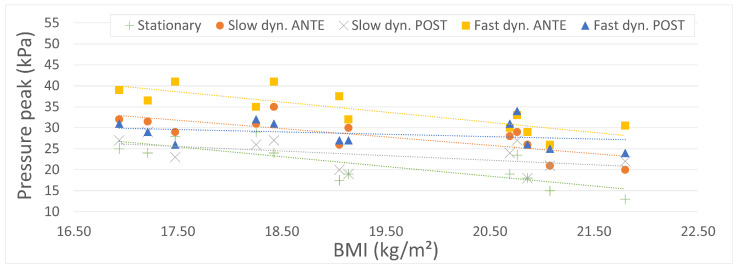
Pressure peaks in the pudendal nerve territory vs. BMI.

Figure 6 summarizes the average pressure peaks extracted from the stationary, slow, and fast dynamic tests carried out on all 12 participants. Each mean value was obtained by averaging the pressure peaks over 10 stabilized cycles, in an attempt to minimize the influence of outliers. In both dynamic tests, anterior and posterior phases are plotted for comparison purposes.

**Figure 7:**
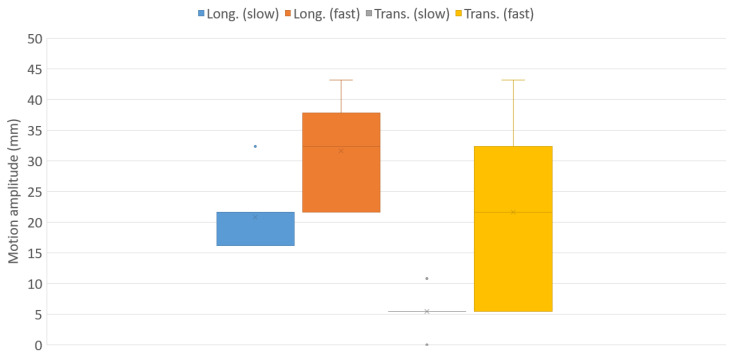
Longitudinal and transverse motion amplitudes of the center of pressure.

Finally, the box plots of the longitudinal and transverse motions of the center of pressure (CoP) are displayed in Figure 7, for the slow and dynamic tests.

## 4. Discussion

As seen in Figure 4, the pressure mappings in all three phases of the simulator motion display a similar distribution, with a pressure peak in the vicinity of the ischial tuberosities. However, the pressure peaks during the anterior phase (i.e., the simulator tilting forward) are clearly shifted forward, and their intensities increase significantly. Indeed, in this phase, the contact between the rider and saddle is no longer ensured through the gluteal muscles, resulting in a thinner layer of soft tissues and an increase in contact pressure, up to values of around 40 kPa. To the best of the authors’ knowledge, no study in the literature mentions a peak pressure threshold for back pain onset in the rider, but Makhsous [22] reports peak pressures of around 23 kPa in workers with chronic low back pain in prolonged sitting positions.

We note from Figure 5 that the mean pressure level in a static situation (simulator halted) is significantly lower than those of the dynamic curves, which is naturally due to inertial effects. Regarding the two dynamic phases, it is fundamental to mention that the amplitudes of the oscillations in the mean pressure peaks are three times higher with the “gallop” setting than with the “canter” setting, with a maximum pressure peak of above 45 kPa. This remarkable difference is due to the vertical acceleration of the simulator in “gallop” setting, which is greater than that of gravity. Although the equestrian’s upper body may not completely be considered as freefalling due to forces exerted by the feet on the stirrups, shocks occur when the simulator goes up while the rider’s body goes down. It was also observed during the test campaigns that the more experienced participants were able to attenuate the intensities of these shocks.

The most remarkable observation to emerge from the analysis of Figure 6 is that all pressure peak plots display similar trends (dotted lines): the higher the BMI of the rider, the lower the pressure peaks. This observation is actually not surprising. Indeed, all participants had very similar heights (mean: 1.66 m, SD: 0.05 m), while their respective masses were quite scattered (mean: 53 kg, SD: 5.86 kg). Even though the associated Body Mass Indexes (BMIs) do not significantly differ (mean: 19.31, SD: 1.68), it can be reasonably assumed that a greater thickness of soft tissues in the area of interest (i.e., pudendal nerve territory) tends to attenuate the pressure peaks resulting from vertical forces exerted by the ischial tuberosities, as mentioned earlier. Not surprisingly, the stationary situation (green) results in the lowest values of pressure peaks, due to the absence of inertial forces. The slow dynamic situation in the posterior phase (grey) does not significantly differ (p=0.21): at an operating frequency of 0.8 Hz, the influence of inertial forces remains limited and the motions of the rider and simulator remain in phase. Moreover, and as mentioned above, the body weight force is transmitted through the gluteal muscles during the posterior phase, which decreases the pressure peaks.

The comparison of anterior (orange) and posterior (grey) slow dynamic plots reveals that the relative angular positions of the rider and simulator result in significant differences in the measured pressure peaks (p=0.01). Pressure mappings in Figure 4 (left and right) clearly depict this discrepancy. A similar conclusion may be drawn in the fast dynamic case (p<0.01). However, it should be noted that the pressure peak values are significantly higher compared to the slow dynamic case (p<0.01) in both the anterior and posterior phases. Actually, increasing the operating frequency (with equal motion amplitude) results in higher inertial forces, causing a phase shift between the relative motions of the rider’s body and the simulator. Consequently, higher contact forces are observed in the fast dynamic tests.

Finally, the motion of the center of pressure (CoP) can be seen as being linked to the tangential displacement between the rider’s pelvis and the saddle, a source of shear stresses in the soft tissues which unfortunately cannot be measured using our experimental setup. It is observed in Figure 7 that as expected, canter allure results in the lowest amplitudes of motion, with very low experimental scattering: the inertial effects remain limited, and all equestrians, regardless of their BMI and experience, are able to maintain a steady position on the simulator. At gallop gait, however, both longitudinal and transverse motions of the CoP display significantly higher mean values and scattering. Quite surprisingly, the transverse motion is much more scattered than the longitudinal one. The reason for this rather contradictory outcome is still not entirely clear, but the participants may not be experienced enough to stabilize their position on the saddle in the transverse direction.

A number of limitations may have influenced the results obtained. First, all experiments were carried out on an electromechanical horse simulator, mostly for simplicity purposes. On an actual horse, the amplitudes and frequencies would not have been constant throughout the tests, resulting in different dynamic pressure peaks. Yet, the present study mostly aimed at proving that the horse gait affects the dynamic pelvic loads, which was demonstrated. Second, only female participants were enrolled in the study, due to the prevalence of pudendal neuralgia in women. Male subjects should also be considered, given the anatomic differences and subsequent changes in perineal pressures. Third, all participants had several years of experience, and a comparison with beginners could prove whether experience and proper technique in riding might be of some preventive benefit in the frame of Pudendal Neuralgia. Finally, further studies should consider the half-seat position, since it involves a periodic loss of contact between the rider and the saddle, which drastically affects the mechanical loads undergone by the perineum.

## 5. Conclusions

To the best of the authors’ knowledge, the present study seems to be the pioneer in the measurement of pressure loads undergone by horse riders. Our findings support the idea that the practice of horse-riding is accompanied by high levels of contact pressure in the perineal area, which are likely to constitute a triggering or aggravating factor for Pudendal Neuralgia (PN). However, the mechanical solicitations (pressure levels and practice frequency) responsible for the appearance of PN in horseback-riding remain unclear, a conclusion that is shared in other sports such as cycling.

Ultimately, future research in this field should come up with sport-wise guidelines that are able to provide, for instance, a maximum daily duration of practice, similarly to the European Directive 2002/44/EC for the exposure of workers to vibrations.

## Data Availability

Not applicable.
